# The 2012 Ming K Jeang award for excellence in cell & bioscience

**DOI:** 10.1186/2045-3701-3-23

**Published:** 2013-05-23

**Authors:** Yun-Bo Shi

**Affiliations:** 1 The National Institutes of Health, Bethesda, MD, USA

## Abstract

Three research groups led by Dr. Lixin Feng of Shanghai JiaoTong University School of Medicine, Shanghai, China, Dr. Walter Wahli of University of Lausanne, Lausanne, Switzerland, and Dr. Gutian Xiao of University of Pittsburgh Cancer Institute, Pittsburgh, USA, won the 2012 Ming K Jeang Award for Excellence in *Cell & Bioscience.*

## Editorial

We are very pleased to announce that three research groups, who each published an outstanding research article in *Cell & Bioscience* in 2012, have been selected to receive the Ming K Jeang Award for Excellence in *Cell & Bioscience*. The Ming K Jeang Award for Excellence in *Cell & Bioscience* was established in 2011 with a generous donation from the Ming K. Jeang Foundation to honor outstanding research articles published in *Cell & Bioscience*, the official journal of the Society of Chinese Bioscientists in America (SCBA; http://www.scbasociety.org). A committee of *Cell & Bioscience* Editors, chaired by Dr. Chris Lau, considered all research articles published in the journal in 2012 to select the three articles to receive the award [1-3]:

PDLIM2 restricts Th1 and Th17 differentiation and prevents autoimmune disease

Zhaoxia Qu, Jing Fu, Huihui Ma, Jingjiao Zhou, Meihua Jin, Markus Y Mapara, Michael J Grusby, Gutian Xiao

*Cell & Bioscience* 2012, **2**:23 (25 June 2012)

Oocyte-like cells induced from mouse spermatogonial stem cells

Lu Wang, Jinping Cao, Ping Ji, Di Zhang, Lianghong Ma, Martin Dym, Zhuo Yu, Lixin Feng

*Cell & Bioscience* 2012, **2**:27 (6 August 2012)

GW501516-activated PPARβ/δ promotes liver fibrosis via p38-JNK MAPK-induced hepatic stellate cell proliferation

Radina Kostadinova, Alexandra Montagner, Erwan Gouranton, Sébastien Fleury, Hervé Guillou, David Dombrowicz, Pierre Desreumaux, Walter Wahli

*Cell & Bioscience* 2012, **2**:34 (10 October 2012)

Congratulations to these three groups of investigators for jobs well done!

We are looking forward to receiving contributions of outstanding research articles from the scientific community in 2013 and beyond.
